# WISP1/CCN4: A Potential Target for Inhibiting Prostate Cancer Growth and Spread to Bone

**DOI:** 10.1371/journal.pone.0071709

**Published:** 2013-08-14

**Authors:** Mitsuaki Ono, Colette A. Inkson, Robert Sonn, Tina M. Kilts, Luis F. de Castro, Azusa Maeda, Larry W. Fisher, Pamela G. Robey, Agnes D. Berendsen, Li Li, Nancy McCartney-Francis, Aaron C. Brown, Nigel P. S. Crawford, Alfredo Molinolo, Alka Jain, Neal S. Fedarko, Marian F. Young

**Affiliations:** 1 Craniofacial and Skeletal Diseases Branch, NIDCR, NIH, Bethesda, Maryland, United States of America; 2 Oral and Pharyngeal Cancer Branch, NIDCR, NIH, Bethesda, Maryland, United States of America; 3 Metastasis Genetics Section, Cancer Genetics Branch, NHGRI, NIH, Bethesda, Maryland, United States of America; 4 Division of Geriatric Medicine and Gerontology, Johns Hopkins School of Medicine, Baltimore, Maryland, United States of America; 5 Department of Oral Rehabilitation and Regenerative Medicine, Okayama University Graduate School of Medicine, Dentistry and Pharmaceutical Sciences, Okayama, Japan; 6 Manchester Immunology Group, Faculty of Life Sciences, University of Manchester, Manchester, United Kingdom; University of Nebraska Medical Center, United States of America

## Abstract

Prostate cancer (PC) is a leading cause of death in men however the factors that regulate its progression and eventual metastasis to bone remain unclear. Here we show that WISP1/CCN4 expression in prostate cancer tissues was up-regulated in early stages of the disease and, further, that it correlated with increased circulating levels of WISP1 in the sera of patients at early stages of the disease. WISP1 was also elevated in the mouse prostate cancer model TRAMP in the hypoplastic diseased tissue that develops prior to advanced carcinoma formation. When the ability of anti-WISP1 antibodies to reduce the spread of PC3-Luc cells to distant sites was tested it showed that twice weekly injections of anti-WISP1 antibodies reduced the number and overall size of distant tumors developed after intracardiac (IC) injection of PC3-Luc cells in mice. The ability of antibodies against WISP1 to inhibit growth of PC3-Luc cancer cells in mice was also evaluated and showed that twice weekly injections of anti-WISP1 antibodies reduced local tumor growth when examined in xenografts. To better understand the mechanism of action, the migration of PC3-Luc cells through membranes with or without a Matrigel™ barrier showed the cells were attracted to WISP1, and that this attraction was inhibited by treatment with anti-WISP1 antibodies. We also show the expression of WISP1 at the bone-tumor interface and in the stroma of early grade cancers suggested WISP1 expression is well placed to play roles in both fostering growth of the cancer and its spread to bone. In summary, the up-regulation of WISP1 in the early stages of cancer development coupled with its ability to inhibit spread and growth of prostate cancer cells makes it both a potential target and an accessible diagnostic marker for prostate cancer.

## Introduction

Being the second leading cause of cancer death in men of all races, prostate cancer is a major health concern for men [1], [2]. It has been proposed that most elderly men harbor traces of prostate cancer, and yet the molecular underpinnings of how and why the cancer progresses are still elusive [3]. Like many other dangerous cancers, prostate cancer cells have a very high incidence of migrating from the primary tumor to distant sites where they are a more direct cause of morbidity and mortality [4]. A frequent site for the metastasis of prostate cancer is to bone, however, when the cancer progresses to this stage, it is usually incurable [5], [6], [7]. Therefore there is a critical a need to 1) understand what factors contribute to the disease progression in the prostate, 2) understand how and why prostate cancers “home” to bone and, further, 3) devise new ways to prevent this complex and devastating process. The metastasis of prostate cancer can be an inefficient process and only a fraction of prostate cancer patients develop cancer that metastasizes to distant sites [3]. By analyzing the early events that take place during prostate cancer progression it is feasible that new diagnostic procedures could be developed that predict the progression and future severity of the cancer and optimize the timing and nature of therapeutic interventions. New information about candidate proteins involved in this process could, also, potentially be used to develop new therapies to reduce the spread and establishment of the disease at distant sites such as bone.

WISP1 (wnt induced secreted protein-1) is a member of the CCN family that is named from its founding members of Cyr61/CCN1, CTGF/CCN2 and Nov/CCN3. There are currently six members of the family that also include WISP1/CCN4, WISP2/CCN5 and WISP3/CCN6 [8]. WISP1 was first identified as a gene expressed in the metastatic melanoma line, K-1735, where it was called Elm1 (referring to its expression in lowly metastatic cells) [9]. Around the same time that Elm1 was discovered, WISP1 was identified in a separate laboratory where it was found to be up-regulated by wnt1 transformed mammary epithelial cells, and in various colon cancer lines as well as being expressed in human colon cancer tissue [10]. Subsequently, WISP1 was shown to confer oncogenic features to rat kidney cells (NRK-49F), including accelerated growth, enhanced saturation density and increased ability to form tumors in mice [11]. Since its original identification, WISP1 has been found in a variety of cancers, including esophageal squamous cell carcinoma [12], chondrosarcoma [13], breast carcinoma [14], [15], neurofibromatosis type I [16], colorectal carcinoma [17], Lewis lung carcinoma [18], invasive cholangiocarcinoma, scirrhous gastric carcinoma [19] and endometrial endometriod adenocarcinoma [20]. Interestingly, WISP1 expression in many of these cancers is localized to the stromal tissues surrounding the cancerous cells [15], suggesting that it could play a role in the microenvironment that supports the growth and/or eventual spread of the primary tumor.

In non-pathological conditions, WISP1 is found in several embryonic and adult tissues that notably include new sites of bone formation [21] where it appears to control osteogenesis [22]. Considering the fact that prostate cancers have a high tropism to bone and, further, that this process can be enhanced by increasing bone turnover [23] we hypothesized that WISP1 may, potentially, be involved in regulating prostate cancer metatasis. This study was undertaken to first determine if and where WISP1 is expressed in primary prostate tumors and then, to determine the role of WISP1 in the growth and homing of prostate cancer cells to skeletal tissue. Our findings showed that WISP1 is found in early stages of prostate cancer either in tissues biopsies or in sera from afflicted patients as well as in the hypoplastic pre-carcinoma tissue from a mouse model of prostate cancer. Furthermore, we showed that inhibition of WISP1 function using neutralizing antibodies reduced the growth of a prostate cancer cell line’s xenograft tumor as well as its homing to bone. Taken together, our study points to WISP1 as a novel participant in the prostate cancer growth and bone metastasis processes.

## Methods

### Ethics Statement

Human Subjects: Core biopsies representing different grades of prostate cancer (from I-IV) including control tissues were purchased from US Biomax™ (Cat#PR801) and their use approved by The National Institutes of Health Office of Human Subjects Research, Bethesda, MD (Exemption #11583). Approval for the use of human serum was obtained from the Johns Hopkins University Medicine (JHM) Institutional Review Board (IRB), Baltimore, MD. The number of the approved IRB protocol for use to assay serum is JHM IRB-X No:04-07-27-03e. The pathological specimens/diagnostic specimens from the commercial suppliers are from sources that are publicly available and the information is recorded by the investigator in such a manner that subjects cannot be identified, directly or through identifiers linked to the subjects and informed consent was obtained from all serum donors prior to its use.

To clarify: the nature of the samples used for human subjects, written consent was obtained from donors as part of the protocols for collecting serum samples (ProMedDx, Inc.) and tissue biopsy cores (US. Biomax Inc.). These protocols and their consent forms were approved by the commercial sources own Institutional Review Boards. Further information about the exact details of the contents of the written approval forms can be found at www.promeddx.com for serum samples and at www.biomax.us for tissue arrays. These protocols and their consent forms were approved by the commercial sources own Institutional Review Boards. Because the commercially available samples are de-identified and de-linked from their donor, their use does not fit the NIH’s definition of Human Subjects Research, therefore the protocols are exempted (Exemption #1158 for the NIH and No:04-07-27-03e for JHU).

### Experiments Using Animals

All procedures using animals were carried out at the National Institutes of health and the institution that granted approval for the animal procedures described in the paper is known as the Animal Care and Use Committee (ACUC). The approval number for the experiments performed in the current study is NIH ACUC #12-645.

### Polyclonal Antibody Production

A human WISP1 peptide with the sequence RDTGAFDAVGEVEAWHRN (amino acids 198–216, accession number NP 003873, see Figure S1A) was synthesized and conjugated through the cysteine to activated keyhole limpet hemocyanin and injected into a rabbit (LF-185) to produce polyclonal antibodies in an AAALAC approved facility (Covance Immunology Services, Denver, PA, USA). The titre of the resultant antiserum was tested using a direct ELISA with either the LF-185 peptide or with purified human WISP1 as control (PeproTec, Rocky Hill, NJ, USA) bound to the microtiter plate. The specificity of LF-185 was tested by Western blot using three other available members of the CCN family Cyr61/CCN1, CTGF/CCN2 and Nov/CCN3 (PeproTec, Rocky Hill, NJ, USA) and showed immunoreactivity only towards WISP1 (Figure S1B). A second rabbit antibody to WISP1 was generated to a sequence at the C-terminus, which is highly conserved (>95%) between mouse and human WISP1 (amino acids 346–367, NP 003873 see, Figure S1A), using the human peptide sequence CRNPNDIFADLESYPDFSEIN conjugated through the cystein to activated KLH (LF-187). This latter antibody also showed high reactivity to WISP1 and not to the other CCN family members tested (S1B).

### Polyclonal Antibody Purification

The peptides used to generate LF-185 and LF-187 were purified using their corresponding peptide and the Sulfolink Immobilization Kit (Thermo Scientific, Rockford, IL, USA), and antibodies were affinity purified as follows. First, the peptide-linked columns were equilibrated according to the manufacturer’s protocol and then 2.0 ml of each serum was passed through the column, washed with PBS, and were eluted with a 4 M guanidine solution in PBS. The eluted antibodies were immediately dialyzed with excess volume of PBS overnight at 4°C and the recovery of their immunoreactivity verified by ELISA. Affinity-purified antibodies were stored at −80°C prior to use. For experimental controls, IgG was purified from a rabbit challanged only with adjuvant using the Protein A IgG Purification Kit (Thermo Scientific, Rockford, IL, USA) with a Protein A affinity column using the same binding, wash, elution and dialysis conditions used for purifying LF-185 and LF-187.

### Immunohistochemistry and TRAP Staining

Slides were stained using the manufacturer’s recommendations in a manner identical to that used for mouse sections described below. Briefly, tissue sections were deparaffinized, endogenous peroxidase activity destroyed by methanolic H_2_O_2,_ rehydrated and incubated with a rabbit polyclonal anti-WISP1 IgG (sc-25441, Santa Cruz Biotechnology, Santa Cruz, CA, USA) diluted to a concentration of 4 µg/mL (1∶50) in PBS plus 10% goat serum overnight at 4°C. A normal rabbit IgG isotype was used as non-immunoreactive control (AB-105-C, R&D Systems, Minneapolis, MN, USA). For some stainings (S5) primary antibodies to human WISP1 (LF-185) or preimmune serum were used and first diluted 1∶500 and incubated at 4°C overnight before detection. Sections were counterstained with Methyl green and areas of positive immunostaining evaluated by at least two independent investigators. For TRAP staining, the TRAP/ALP stain kit (#294-67001, Wako, Osaka, Japan) was used following the manufacturers recommendations.

### Serum Analysis

Serum from patients with defined grades of prostate cancer or from normal controls was resolved by SDS-PAGE, transferred to nitrocellulose and probed with a polyclonal antibody against the conserved carboxy-terminus of human WISP1 (LF-187) by standard Western blotting procedures. The primary antibody, LF-187, was added at a dilution of 1:2000 followed after incubation and washing with a HRP-labeled secondary antibody used at a dilution of 1∶10,000. Following removal of second antibody solution, the membrane was washed and exposed to the chemiluminescent enzyme substrate and signals were captured, digitized and analyzed using a Kodak GEL Logic 2200 Imaging System (Carestream Health Inc., Rochester, NY, USA). For each blot, the net intensity of the band corresponding to full-length WISP1 was normalized to the average signal from duplicate lanes containing 20 ng of recombinant WISP1 (PeproTec, Rocky Hill, NJ, USA).

### Animals

Immunocompromised mice (athymic nude-Foxn1nu) were purchased from Harlan and were 8 weeks of age at the time of the study. A mouse model of prostate cancer (transgenic adenocarcinoma mouse prostate or TRAMP) that over-expresses the simian virus 40 (SV-40/Tag) gene from the prostate specific probasin (PB) promoter strain was previously generated [24] and used to examine WISP1 expression in affected prostate tissue. The genetic background of the TRAMP mice was NOD and was obtained by crossing the TRAMP B6 females with NOD/LtJ males to generate an F1 strain hemizygous for the PB-Tag. Resulting TRAMP-NOD mice were euthanized by pentobarbital overdose at 8 and 16 weeks of age and prostate and seminal vesicles harvested for histological analysis. Tissues were fixed in buffered formalin overnight and then transferred to 70% alcohol. Fixed tissues were embedded in paraffin and sectioned to a thickness of 4 µm. To determine the relative bone mineral density of the ant-WISP1 treated mice we used a Lunar PIXImus Densitometer (GE Medical Systems) specifically designed for rodents.

### Colonization of PC-3 Luc Cells to Distant Sites by Intracardiac Injection (IC)

Eight week-old male Athymic nude- NIH/Bg-nu/nu-XID mice were anesthetized with isofluorane and shaved at the injection site that was then swabbed with iodine and 70% alcohol. A TB syringe with a 27 gauge needle attached was then loaded with a suspension of PC3-Luc (5×10^5^ cells/150 ml of PBS) and the needle inserted vertically in the fourth intercostal space. When a flash of blood was noticed in the hub of the needle the cells were injected slowly until the contents of the needle were empty and the needle was pulled out slowly. To assure ourselves that the cells were correctly injected into the left ventricle and disseminated throughout the mouse and not, for example, trapped in the lungs (as would be the case for an injection into the right ventricle) after IC injection the mice were immediately injected with 100 µl of a 40 mg/ml solution d-Luciferin firefly (Biosynth) intravenously and imaged using a Lumina-XR (Caliper Life Sciences, Hopkinton, MA, USA). Mice that showed distribution of the PC3-Luc cells as judged by luminescence throughout the entire body were then used for further treatments. The mice were divided into three treatment groups (n = 6/group) and injected with cells on Day 0. Administration of antibodies was carried out with a dose of 100 µg via IP injection twice per week, starting on Day 0 of tumor inoculation and continuing throughout the experiment. Three groups consisted of: 1) mice injected with 100 µl affinity purified antibody, LF-185, 2) those given control IgG, and 3) those given 1X PBS alone. *In vivo* imaging was performed weekly to track the growth and establishment of tumor cells using a Lumina-XR (Caliper Life Sciences, Hopkinton, MA, USA). Prior to imaging mice were injected with 100 µl of a 40 mg/ml solution d-Luciferin firefly (Biosynth) in PBS intravenously. Mice were treated for a total of 4 weeks and the area and counts of light exposure were quantified as described above. In the fourth week, after the final luciferase analyses, the bones exhibiting colonization by the tumor cells were harvested and analyzed using an MX-20 Faxitron radiography system with exposure at 30 Kv for 40 seconds using PPL film from Kodak with subsequent embedding and processing for histology and immunochemistry.

### Cell Culture and RT-PCR

PC3-Luc cells [25] are a human prostate cancer constitutively expressing luciferase (a gift from Dr. Russell Taichman, University of Michigan School of Dentistry, USA). Cells were cultured in RPMI Medium 1640 (Invitrogen, Grand Island, NY, USA) containing 10% FBS (Atlanta Biologicals, Lawrenceville, GA, USA) and 1% penicillin/streptomycin solution (Gibco GlutaMAX-I, Grand Island, NY, USA) in a 37°C atmosphere of 5% CO_2_/air. Cells were passaged every 2 days at 85% confluence. RT-PCR was performed on mRNA isolated from cultured PC3-Luc cells and amplified using oligonucleotide sets corresponding to human WISP1 using sequences and conditions described previously [22].

### Xenograft

Human prostate cancer cells PC3-Luc were delivered to 8 week old athymic nude-Fox1nu mice by subcutaneous inoculation of a tumor cell suspension (5×10^6^ cells/200 µl of PBS) on day 0. In this experiment cells were counted, resuspended in 200 µl of cold PBS and kept in sterile tubes on wet ice during transport to the animal facility. The optimal number of cells for this experiment was determined by first measuring overall PC3-Luc growth using 2×10^6^, 5×10^6^ 1×10^7^ cells/inoculation on 4 different sites/mouse. For the inoculation, cells were drawn into a TB syringe with a 25G needle attached and injected with bevel side up into the dorsal side previously wiped with alcohol. The size of the tumor was then measured by caliper or by relative luciferase activity using the Lumina XR as described above. Our pilot study showed that the lowest 2×10^6^ dose of PC3-Luc grew slowly, while the highest dose 1×10^7^ dose grew very rapidly both of which we judged to be sub-optimal for the 4-week time course planned for our antibody treatments. The middle dose of 5×10^6^ was optimal and then used for subsequent experiments. Three treatment groups comprised of 6 challenged nude mice per group injected intraperitoneally (IP) twice a week with either 1) 100 µg of affinity-purified polyclonal antibody directed against WISP1 (LF-185) 2) 100 µg of similarly purified control IgG or 3) PBS alone in a volume of 100 µl. Freshly prepared purified antibodies were prepared prior to injection into the test mice. Through the course of the experiments tumors were measured with a caliper to estimate their relative growth rate and in the fourth week, the tumors were harvested and weighed. In one experiment mice were followed for 6 weeks (S4) with similar results. All experiments using mice were repeated at least twice. At the end of the experiment some tumors were further analyzed by histology and analyzed for WISP1 expression as described in the previous section “Immunohistochemistry and TRAP staining”.

### In vitro Migration

*In vitro* migration of the PC3-Luc cells was tested using BD Falcon FluoroBlok Cell Culture Inserts with 8-micron holes (BD Biosciences, Bedford, MA, USA). PC3-Luc cells in logarithmic growth phase were detached from 100 mm plates by trypsin-EDTA, and 2×10^4^ cells were pretreated with 100 µg/ml of LF-187, IgG antibody or PBS for 1 hour at 37°C in serum free RPMI 1640 and were added onto FluoroBlok Cell Culture Inserts. RPMI 1640 containing 5% FBS or 200 ng/ml WISP1 (PeproTec, Rocky Hill, NJ, USA) was added to the lower chamber, and the entire system was incubated at 37°C for 24 hours in 5% CO_2_. After incubation and fixation, cells were stained with DAPI (cat# P36935, Molecular Probes, Life Technologies, Grand Island, NY, USA). Migrated cells were examined microscopically on the lower side of the membrane by detecting migrated DAPI-labeled nuclei. The number of cells in 3 random whole fields at 100x magnification was counted with Image-Pro Plus (MediaCybernetics, Rockville, MD, USA) and the average of three wells was determined.

### Statistical Analysis

An unpaired Student’s *t*-test was used to compare control vs. experimental samples using GraphPad Prism software (Prism 5, GraphPad Software, Inc., La Jolla, CA, USA) for cell migration, tumor growth and luciferase assays. *P* values <0.05 were considered statistically significant. The distribution of measured parameters for serum WISP1 values was assessed by the D’Agostino & Pearson omnibus normality test. Comparisons between two groups were performed using an unpaired Student’s *t*-test, while comparisons across three or more groups utilized a one-way ANOVA test. The association of WISP1 protein levels with PSA was assessed using a Spearman correlation. *P* values <0.05 were considered statistically significant.

## Results

### WISP1 Protein Expression in Prostate Cancer Tissue and in Serum from Affected Patients

**Figure 1:**
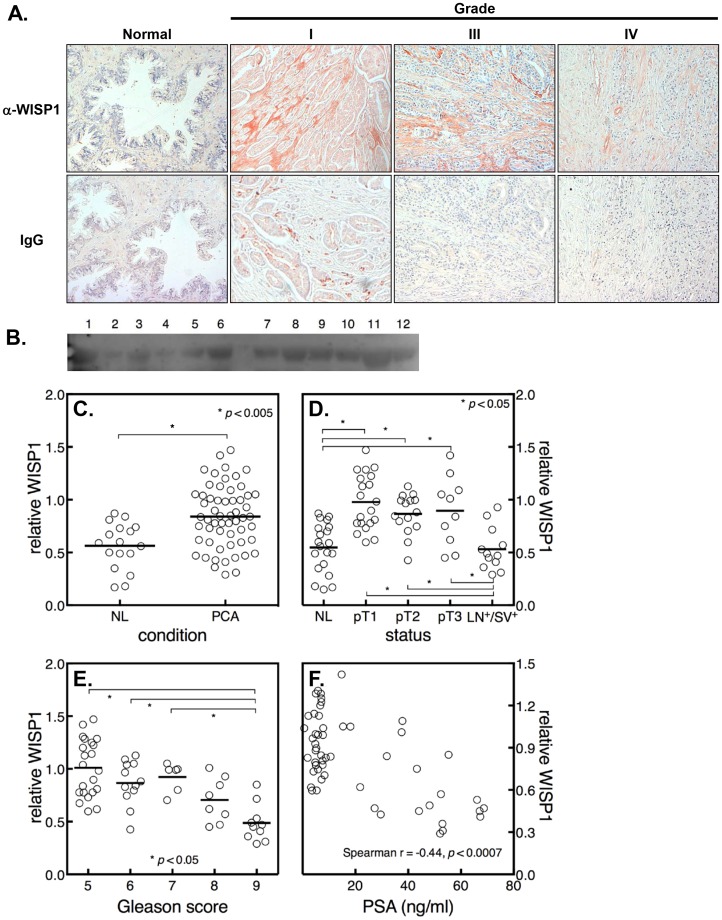
WISP1 expression in prostate cancer tissue and in serum from affected patients. **A.** Immunohistochemical detection of WISP1 in biopsies from patients with various grades of prostate cancer. Specific staining for WISP1 (LF-185) was found in the stroma and in the epithelium of tumors from lower Gleason Grade (Grade I) scores and diffusely throughout the highly metastatic (Grade IV) neoplastic carcinoma. **B**. A representative image of a quantitative western blot assay used to measure serum levels of WISP1 in serum from normal subjects (lanes 1–6) and from subjects with prostate cancer (lanes 7–12). **C.** Quantified bands from 20 normal subjects and 60 subjects with prostate cancer were compared by *t*-test. **D.** Samples were stratified by disease stage using the TNM scoring system and compared by an ANOVA test. NL, Normal, pT1, pT2, pT3 lowest to the highest severity, LN^+^/SV^+^, lymph node positive, seminal vesicle positive. **E.** The levels of WISP1 were stratified by Gleason scores and compared by ANOVA test, 5 is lowest severity, 9 is greatest severity. **F.** The association of serum WISP1 levels with PSA was analyzed by Spearman correlation (PSA, X-axis, WISP1, Y-axis). Each small circle represents values from a single patient.

Antiserum were raised against a poorly and highly conserved domain of WISP1 (LF-185 and LF-187 respectively, see S1) in rabbits and found to be highly reactive to WISP1 (S1) and unable to cross-react with three other members of the CCN family including Cyr61/CCN1, CTGF/CCN2 or Nov/CCN3 (S1). Although not directly tested by Western blot, human WISP2/CCN5 and WISP3/CCN6 do not contain any primary protein sequences that could reasonably be considered to be homologous to the highly conserved human peptide used to produce LF-185. The location and relative level of expression of WISP1 in normal human prostate and prostate cancers of various degrees of severity was assessed with LF-185 using tissue core biopsies commercially obtained from Biomax™. In this experiment core biopsies from low and high grade cancers (I was lowest, IV was highest) and were stained for immunohistochemistry using the WISP1 antisera and compared with staining using IgG as a negative control. Evaluation of this staining by at least two independent observers revealed that WISP1 is expressed to a greater extent in the prostate cancer compared to normal controls (Figure 1A) and that it was primarily located in the stroma tissue surrounding the tumor and to some extent in the epithelial tissue. In addition to this, WISP1 staining was higher in samples that were from patients with the lowest grade of cancer (I) compared to those with the higher grades (IV) of cancer.

Our immunohistochemistry data showing that WISP1 expression was strongest in lower grade biopsies led us to speculate that WISP1 in such cancers could escape into serum and be detected using immunoblotting techniques. To test this hypothesis, serum samples were examined by western blotting using antibodies to WISP1 (Figure 1B). Our analysis showed that when all the grades were grouped together WISP1 was significantly higher in serum from patients with prostate cancer (PCA) compared to normal controls (NL) (Figure 1C). These samples were further subdivided using the tumor, node, metastasis (TNM) scoring system and showed that the relative levels of WISP1 were significantly higher in patients with grades pT2, pT2 and pT3 compared to either NL or to patients that were lymph node and seminal vesicle positive (LN+/SV+) (Figure 1C). As predicted, when the samples were re-evaluated using the Gleason scoring system the relative levels of WISP1 were significantly higher in grades 5–7 compared to the more advanced grades 8–9 (Figure 1E). Interestingly, when compared to PSA levels there was a significant inverse correlation with WISP1 being highest in the samples that had the lowest PSA levels (Figure 1F).

### WISP1 Expression in the TRAMP Model of Prostate Cancer

**Figure 2:**
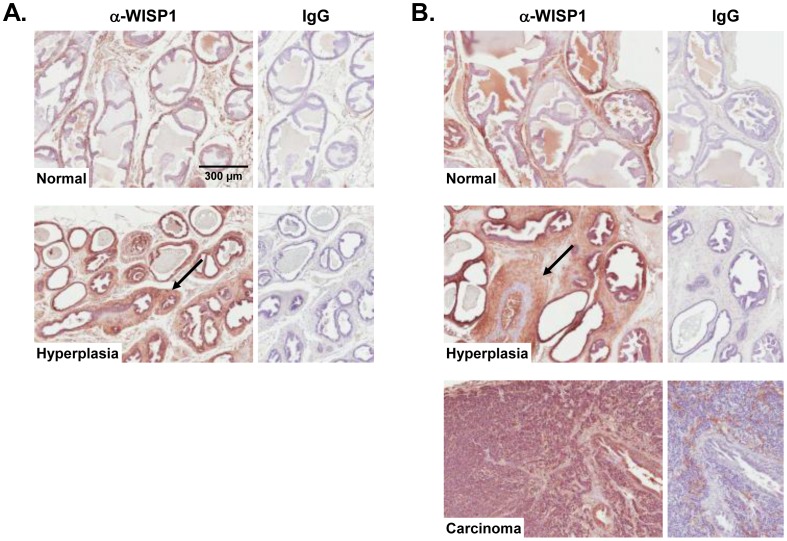
WISP1 immunohistochemistry of prostate tissue from TRAMP mice. **A.** WISP1 localization at 8 weeks of age in normal prostate tissue (upper panel) and hyperplastic tissue (lower panel, black arrow). **B.** WISP1 localization at 16 weeks of age in normal prostate tissue (upper panel), hyperplastic tissue (middle panel, black arrow) and carcinoma (lower panel). IgG controls are shown to the right of each panel.

In order to confirm our notion that WISP1 was up-regulated in prostate cancer we used a previously generated mouse model known as TRAMP (transgenic adenocarcinoma mouse prostate). This strain of transgenic mice begins to acquire hypoplastic prostate tissue by 8 weeks of age and by 16 weeks the affected tissues progress to carcinomas with significant tumor burden. Affected prostates were isolated from TRAMP-NOD mice at early (8 weeks of age) and late (16 weeks of age) stages of tumor progression and analyzed for WISP1 expression by immunohistochemistry. In the early stages of the disease WISP1 was highly expressed in the hypoplastic tissue that initially develops in the model at that age (Figure 2A bottom panel). In tissues isolated from 16 week-old mice, WISP1 expression was increased even more with the further development of hyperplasia (Figure 2B middle panel) and, further, was expressed broadly in regions with advanced carinoma (Figure 2B bottom panel).

### Anti-WISP1 Treatment Reduces the Spread and Establishment of PC3-Luc Distant from the Site of Injection

**Figure 3:**
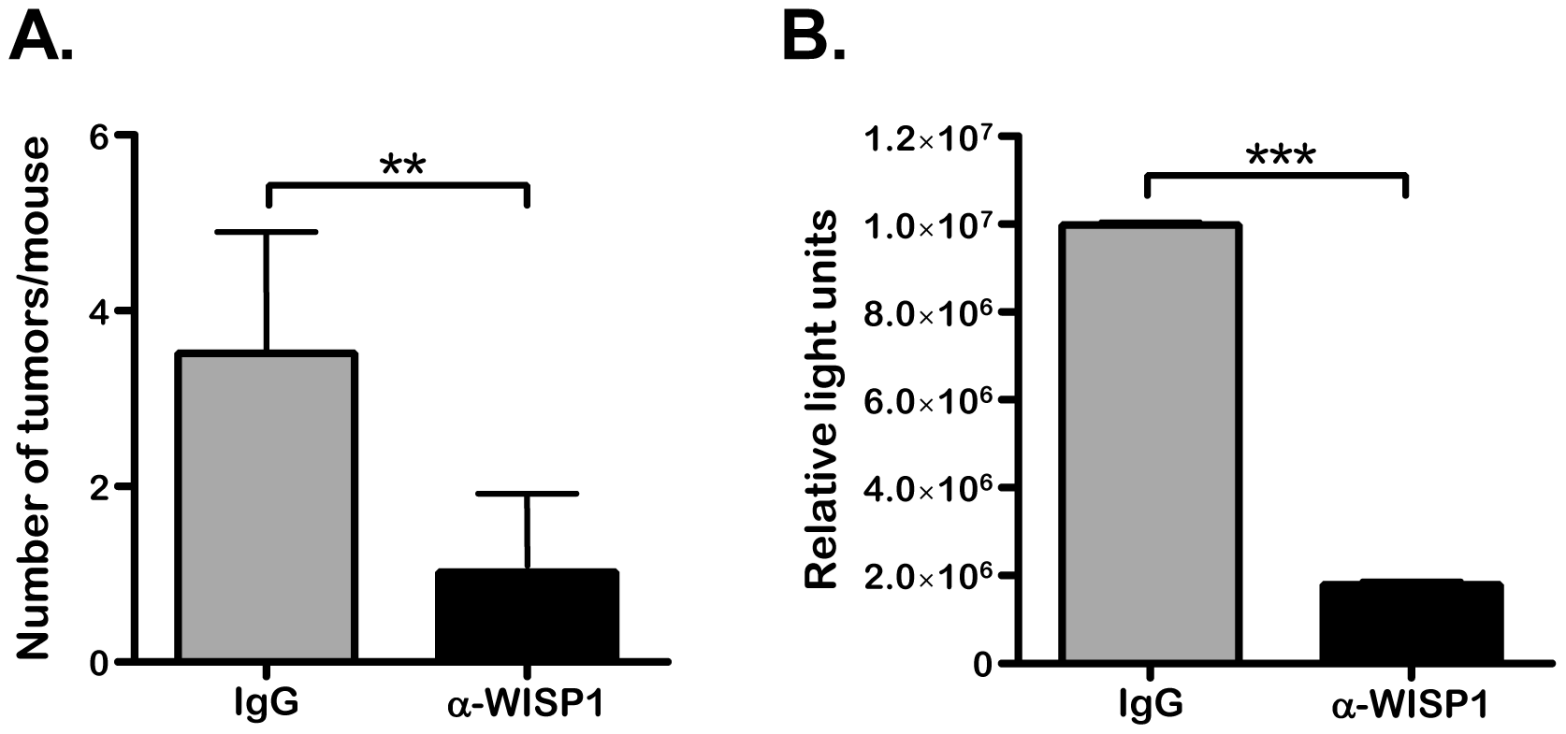
Treatment with WISP1 antibodies reduced spread and establishment of PC3-Luc tumors in immunocompromized mice when injected IC. A. Total number of tumors developed per mouse treated with IgG or anti-WISP1 (LF-185). **p<0.01 PBS vs. anti-WISP1 treatment, B. Total tumor burden judged by relative luciferease measurements per mouse. ***p<0.001 IgG vs. anti-WISP1 treatment.

PC3-Luc cells were injected into the left ventricle of immunocompromized mice and allowed to spread and grow for 4 weeks with twice weekly injections of the PBS, IgG or anti-WISP1. The number of tumors and total tumor burden were then determined by their ability to emit light (luminescence) and showed that anti-WISP1 significantly reduced both of these parameters compared to PBS or IgG treatments (Figure 3A and 3B respectively). In our hands the PC3-Luc cell line used in the study had a high triopism for bone making its way after IC injection to numerous skeletal sites including the jaw, snout, spine, femur, tibia, ribs, sternum, scapular and ulnae. However, other sites of PC3-Luc cell homing were also found outside the skeleton and included soft tissues such the heart, lung and testicle comprising approximately10% of all the metastases. A representative picture of the tumors formed after IC injection is shown in (S2) that illustrates these findings. When the numbers of tumors were counted we found that the skeletal sites were preferentially decreased by the WISP1 antibody treatment. Specifically, in controls, 90% of the metastases were in hard tissue and 10% in soft tissue. With WISP1 antibody treatments the number of skeletal sites affected was reduced to 66% of the total metastases with the soft tissue sites making up 33% of the total metastases. In addition to total tumor number the total tumor burden was reduced in by WISP1 antibody treatments compared to IgG treatments (Figure 3B). This indicated that not only dissemination but also tumor growth was reduced by WISP1 neutralization. To test the possibility that WISP1 could affect tumor grown we next evaluated growth of the PC3-Luc using xenografts.

### WISP1 Antibody Treatment Reduces the Size of PC3-Luc Xenografts in Immunocompromised Mice

**Figure 4:**
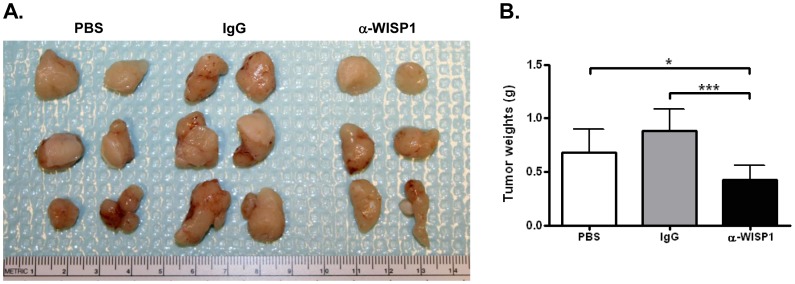
Growth of PC3-Luc human prostate cancer cell sub-cutaneous xenografts were reduced by treatment with antibodies specific to human WISP1. **A**. Photograph of representative PC3-Luc tumors dissected from immunocompromized mice treated with PBS (left), IgG (middle) or anti-WISP1 (LF-185, right). **B**. Weight in grams of PC3-Luc cell tumors grown in mice treated with PBS, IgG or anti-WISP1 (LF-185) after 4 weeks. *p<0.05 PBS vs. anti-WISP1, ***p<0.001 IgG vs. anti-WISP. PBS vs. IgG were not significantly different.

Antibodies against WISP1 were used along side IgG and PBS controls to test their relative ability to reduce the growth of prostate cancer cells that were grown under the skin of immunocompromised mice. Animals were injected twice a week with anti-WISP1 and tumor growth monitored for 4 weeks. Considering that WISP1 is made in bone we also examined the mice using a DEXA scanning machine and showed that anti-WISP1-treated mice had no significant changes in the percentage of bone mineral density before and after treatment compared to either PBS or IgG controls (S3). The rate of tumor growth was measured during the course of the experiment using calipers and indicated that the tumors in the mice treated with anti-WISP1 were reduced in size compared to either PBS or IgG controls (S4). When the tumors were removed at the end of the treatments and photographed, it was clear that tumors were smaller in the anti-WISP1 treated mice, (Figure 4A) and that they had statistically less overall weight compared to tumors from mice treated with either PBS or IgG (Figure 4B).

### Appearance and Expression of WISP1 in PC3-Luc Tumors Invading Bone

**Figure 5:**
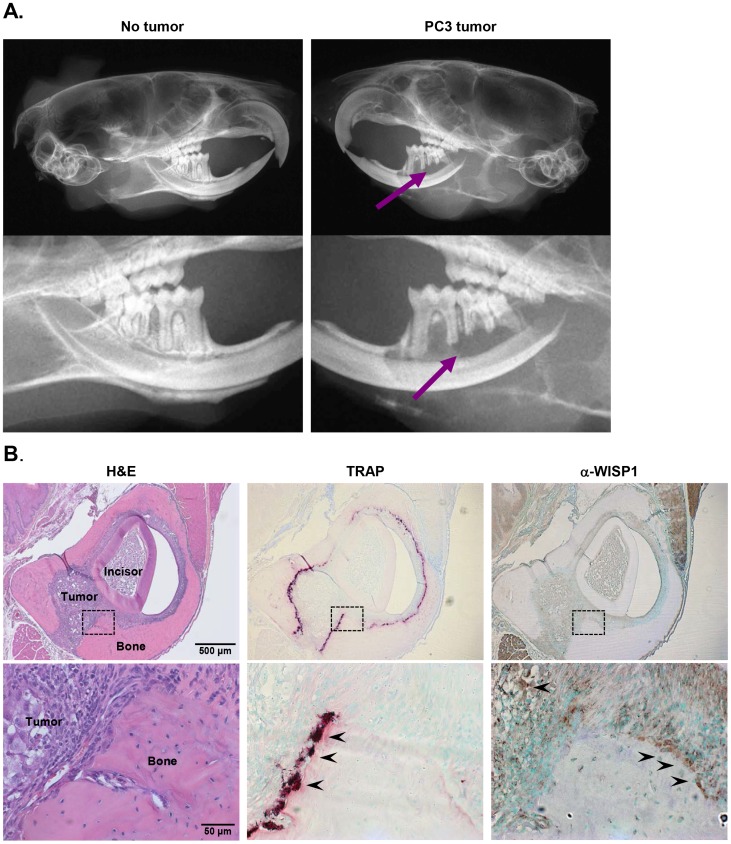
PC3-Luc tumor spread and establishment. **A.** X-ray of sagittal view of a head from a control mouse devoid of luciferase/PC3-Luc cells (left panel) compared to the head of a mouse with an established tumor (right panel). The lower panels are higher power images of the images shown above them with the arrows pointing to the site of tumor integration below the molars that is more radio-opaque compared to the control. **B.** Histological sections through a regions of the jaw showing tumor invasion within the bone tissue just below the tooth (incisor). Left, Hematoxylin and eosin stain, middle, staining with TRAP, right immunohistochemistry using anti-WISP1 to show the location of WISP1 relative to the osteoclasts. Upper panels are 50X and the boxed area is shown in the lower panels is 430X magnification. Arrow heads point to the TRAP stained osteoclasts (middle panel), and the localization of WISP1 (brown stain) in the tumor and at the bone tumor/PDL interface.

The nature of the PC3-Luc tumors formed 4 weeks after IC injection was first assessed by light emission in live mice using a Lumina-XR, system which was done to determine their precise location for further processing. As previously noted [25], this line of PC3-Luc cells has a high tropism to bone, and in particular appears to “home” to sites within the alveolar bone in the jaw just below the teeth (Figure 5A, arrow). To determine the location and WISP1 expression in the invading tumor and its proximity relative to the osteoclasts that were actively resorbing the bone tissue in this lytic tumor model we prepared sections through a tumor in the jaw and stained them for Tartrate-resistant acid phosphatase (TRAP) an enzyme enriched in the osteoclast and for WISP1 by immunohistochemistry. As predicted, the border of the tumor surrounded by bone was rife with osteoclasts judged by the pattern of TRAP staining (Figure 5B, middle panel). Serial sections subject to immunohistochemistry using WISP1 antibodies showed that WISP1 was also expressed (compared to IgG controls) at the interface between the alveolar bone and the invading PC3-Luc tumor (Figure 5B, arrow) however it was notably located in a position near but not coincident with the TRAP positive osteoclasts (compare middle and right panels, Figure 5B). WISP1 was also found within the invading tumor (Figure 5B). Sections trough a control, non-tumor bearing jaw showed a low level of both WISP1 and TRAP expression in normal bone and in the periodontal ligament surrounding the tooth (not shown).

To determine the source of WISP1 that is being blocked by the WISP1 antibody treatments we carried out immunostaining using antibodies to WISP1 and showed that xenografts composed of PC3 tumor cells contained some WISP1 judged by the relative intensity of staining using LF-185 vs IgG control (S5A). When PC3 cells were grown *in vitro* and protein extracted we also found of WISP1 present (not shown). Finally, to determine if WISP1 mRNA is being translated in the PC3 cells we extracted mRNA and using oligonucleotides specific for WISP1 and detected an amplicon of the correct size (S5B) indicating to us that the WISP1 found in PC3 cells both *in vitro* and *in vivo* could come, at least in part, from the PC3 cells themselves. This was further verified using sections of the PC3-Luc cell tumor within the jaw; WISP1 staining was found in both the tumor and in regions outside the tumor at sites of bone remodeling and in the PDL (Figure 5B) near but not within the osteoclasts.

### Migration of PC3-Luc Cells was Blocked by Treatment with WISP1 Antibodies

**Figure 6:**
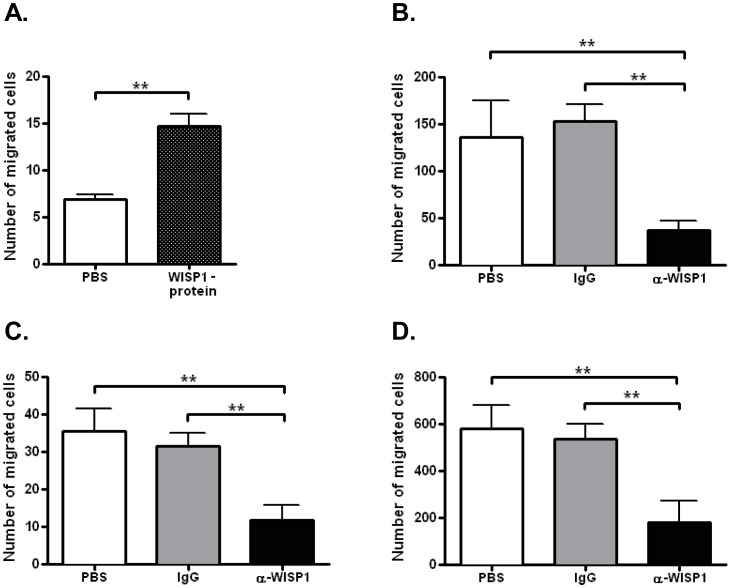
Cell migration and invasion is blocked by treatment of PC3-Luc with anti- WISP1. **A.** Chemotaxis of PC3-Luc cells towards the bottom of a Boyden chamber containing WISP1. **p<0.01 PBS vs. WISP1-protein. **B.** Relative chemotaxis levels of PC3-Luc pre-treated with PBS, IgG or anti-WISP1 (LF-187) towards a gradient of 5% FBS. **C.** Relative chemotaxis levels of PC3-Luc pre-treated with PBS, IgG or anti-WISP1 (LF-187) towards 200 ng/ml WISP1 protein. **D.** Relative invasion of PC3-Luc cells treated with IgG, PBS, or anti-WISP1 (LF-187) through membranes coated with Matrigel™ toward a lower chamber containing WISP1-protein.

Considering the fact that WISP1 is concentrated at the bone-tumor interface and that the PC3-Luc cells “home” to bone we wondered whether WISP1 could have chemotactic properties. To test the chemotactic capacity of the PC3-Luc cells they were placed in a Boyden chamber with varying concentrations of FBS above and below the membrane they traverse through (S6). Significant migration of the PC3-Luc cells towards 5% FBS in the bottom chamber from serum free media in the top chamber was observed indicating the cells indeed possessed chemotactic abilities. When the experiment was repeated using WISP1 protein as chemoattractant the migration of PC3-Luc cells across the membrane was significantly increased compared to a PBS control (Figure 6A). To test the ability of anti-WISP1 to block these chemotactic activities the tumor cells were pre-incubated with either anti-WISP1, IgG or PBS prior to migration. Anti-WISP1 treatment significantly blocked the migration of PC3-Luc towards media containing 5% FBS (Figure 6B) or WISP1 (Figure 6C) compared to either PBS or IgG controls. Finally, the invasion of PC3-Luc cells across membranes coated with Matrigel™ was tested and showed that anti-WISP1 inhibited PC3-Luc invasion towards the bottom chamber containing WISP1 to a much greater extent than cells treated with either IgG or PBS.

## Discussion

The goal of our investigation was to determine whether WISP1 could play a role in prostate cancer growth and spread to bone and then to provide evidence that it could be a novel target for detection and future therapeutics. Using antibodies against WISP1 we found they could block the growth of xenografts and the localization of PC3-Luc prostate cancer cells to bone. One way that anti-WISP1 treatment might inhibit cancer growth and spread could be by controlling cancer cell migration. In this paper we showed that PC3-Luc cell migration towards increased concentrations of fetal bovine serum (FBS) or to purified WISP1 could be blocked by pre-treating the cancer cells with WISP1 antisera. Exactly how WISP1 controls cell movement is not known, but is likely to involve interaction with one or more integrins. We recently reported that WISP1 regulates the binding of BMP-2 to bone marrow stromal cells (BMSCs) using a mechanism that depended on the integrin α5 [22], [26]. Whether similar interactions are taking place in PC3-Luc cells as they migrate towards bone is not clear and will need to be addressed in future studies. Whatever the mechanism, it is logical to propose that one reason PC3-Luc cells are attracted to bone is because of the high levels of WISP1 found there [26] which could, in turn, lead to what is now referred to as a “fatal attraction” [5].

It is also not yet known which of the several domains of WISP1 are important for PC3-Luc migration, invasion and homing. To date, at least 6 different transcripts of WISP1 have been identified in the NCBI Nucleotide database (http://www.ncbi.nlm.nih.gov/sites/entrez?db=pubmed) that are composed of various combinations of the structural domains referred to as IGF binding protein (IGFBP), Von Willebrand factor type C (VWF), thrombospondin1 (TSP1) and the cysteine rich terminus (CT). One WISP1 variant known as WISP1v lacks the VWF domain and is highly up-regulated in Scirrhous gastric carcinoma [19], and, when ectopically expressed in cultured cells, causes them to have a more invasive phenotype. WISP1v is also expressed by human bone marrow stroma cells (hBMSCs), where it responds differently to TGF-β induced proliferation compared to full length WISP1 [27]. It will be interesting to determine the precise location of WISP1v and the other WISP1 variants and, then, to elucidate if they have unique, overlapping, or even competitive functions in controlling prostate cancer cell function.

Immunohistochemistry of the PC3-Luc xenografts as well as RT-PCR of mRNA extracted from PC3-cells grown *in vitro* show the presence of WISP1 protein and mRNA respectively. In spite of this finding we can not exclude the possibility that WISP1 also comes from the bone, the circulation or both. Work from our lab and others shows that WISP1 can be extracted in substantial quantities from demineralized bone [26] and can be produced by differentiating bone marrow stromal cells [22], [27] and, further, that it is found at sites of new bone formation [21]. In this regard it is important to note that the PC3-Luc cells used in this experiment that migrated and established themselves in bone are lytic causing the bone to dissolve (Figure 5). Thus, the expression of WISP1 at the bone tumor interface could be coming from the PC3 cells, the resorbing bone, and either made by the osteoblasts or by the osteoclasts. To further address this question histological sections were prepared and magnified at this interface and stained with both WISP1 and TRAP, a marker for osteoclasts. Our data showed that WISP1 localizes at the sites of resorption near but not within in the osteoclast. In summary, since WISP1 is made by PC3 cells and by bone forming cells we conclude that the source of WISP1 could come from either the osteoblast that produces the WISP1 found in bone, the PC3 tumor that have both detectable mRNA and protein or from both. Whatever the source it is clear that blocking WISP1 function inhibits the journey and establishment of PC3 tumor cells in bone. Additional experiments using siRNA in PC3 cells will be needed to further resolve this point.

In addition to its expression at the bone-tumor interface, WISP1 is predominant in the stroma tissue surrounding the primary prostate tumor cells. Considering these distinct localization patterns, we suspect that WISP1 could be a “bi-directional” [6] link in the communication between the prostate cancer and the surrounding microenvironment. In this context, WISP1 could potentially serve to enrich the cancer cell milieu, subsequently facilitating cancer cell activities such as cell migration, and cell growth where the cancer cells themselves also contribute to changes in the microenvironment [28]. Such interaction might then accelerate the “vicious cycle” in cancer metastasis [29], caused by perturbations in the connections between the cancer cells (seed) and the surrounding stroma (soil) [30]. We show that WISP1 levels in the primary prostate cancer stroma and in the serum from patients afflicted with this disease decreases with increasing severity of the cancer. In this regard it can be noted that one hallmark of cancer progression is the induction of proteases that presumably cause the destruction of surrounding tissues aiding the cancer cells to make their way out of the primary tumor site to distant locations. Such proteases could degrade WISP1 such that its abundance in both the primary tumor and in the serum from afflicted patients is reduced. Further experiments that examine the level and activity of proteases to see if they preferentially target WISP1 are needed to fully understand how and why the induction of WISP1 in the prostate tumor eventually recedes as the prostate cancer reaches advanced stages.

Several other CCN family members besides WISP1 have also been implicated in cancer. Cyr61/CCN1 has been linked to both skeletal (osteosarcoma) [31] and prostate cancer [32], [33]. In prostate cancer, the expression of Cyr61/CCN1 is associated with lower risk of disease recurrence [34], and its expression is highest in prostate tumor cells that have low levels of p53 tumor suppressor [32]. Cyr61/CCN1 is also expressed in pancreatic cancer [35] and in human chondrosarcoma cells where it appears to up-regulate MMP13 expression and cell migration [36]. CTGF/CCN2 is linked to breast cancer metastasis, where it regulates angiogenesis [37] in a manner that could be further influenced by PTHrP. Nov/CCN3 is differentially expressed in human prostate cancer cell lines and tissues [38], where it is specifically localized to epithelial tissue. WISP3/CCN6 expression is linked to the severity of breast cancer and is implicated in regulating the epithelial to mesenchymal transition (EMT) [39]. Taken together, it is possible to imagine that CCN specific antibody interference could also be used to both diagnose and treat the numerous cancer types where they are expressed. Promising pre-clinical studies showing inhibition of metastasis of breast [37] and pancreatic cancer using antibodies to CTGF/CCN2 [40] further validate this concept.

Many theories abound about the potential role of the EMT in cancer progression. During this process, the ordered alignment and shape of epithelial glandular cells, as well their characteristic gene expression patterns, “transition” to become more mesenchymal –like, being less adhesive and more dysmorphic. The role of WISP1 in regulating the EMT process accompanying idiopathic pulmonary fibrosis (IPF) has recently been investigated in a mouse model with IPF induced by bleomycin treatment [41]. When the diseased mice were treated with antibodies against WISP1, the EMT and subsequent fibrosis were reduced, causing the mice to live longer than their untreated counterparts [41]. In light of this new finding, it is tempting to speculate that anti-WISP1 treatment could, in a similar fashion, modulate the EMT in prostate cancer. In this case, it is likely that the supporting stroma where WISP1 is expressed will be one important factor in regulating the activities of the transforming prostate cells. In this context, many consider the stroma to be a key target for cancer therapy because of its important roles in regulating the cancer cell microenvironment and ultimately cancer cell fate [28], [42].

The accessory molecules that could modulate WISP1’s functions in prostate cancer cell are not entirely clear, however, considering what is known about WISP1 in normal tissues, it is possible that the BMP/TGF beta family members are somehow involved. In hBMSCs WISP1 inhibits TGF-β1 induced Smad2 phosphorylation as well as TGF-β1 induced proliferation of BMSCs [26]. BMP-2-induced Smad1 phosphorylation and subsequent osteogenesis, on the other hand, is enhanced by WISP1 [22]. When BMP-2 action is down-regulated by treatment with the BMP antagonist, noggin, PC3 cells have less migration and invasion *in vitro* and less tumor formation *in vivo* [43]. In this regard, it is possible that WISP1’s positive influence on BMP-2 could be one way it increases migration, invasion and spread of prostate cancer to bone. The positive influence of WISP1 on BMP-2 could therefore be part of the molecular underpinnings related to increased PC3-Luc metastasis in mice that have increased bone turnover induced by intermittent application of PTH [23]. BMP-7 has also been implicated to control prostate cancer growth and spread, however, its potential relationship to WISP1 remains to be clarified [44], [45]. One other factor that may be connected to WISP1 function is vitamin D_3_ an agent known to be beneficial to bone and in reducing cancer. The WISP1 promoter has numerous vitamin D_3_ responsive elements and pilot work from our lab shows it is down regulated in hBMSCs treated with 1,25 dihydroxyvitamin D_3_ (unpublished data). Our future challenges will be to confirm and identify new connections that tie WISP1 function to cancer and bone.

Interestingly, WISP1 expression is itself up-regulated by both TGF-β1 and BMP-2 [46], suggesting a “feed forward” regulatory loop for the control of these growth factors known to be important for both bone and cancer regulation. It is possible the coupled interference of TGF-β1 and BMP-2 by anti-WISP1 treatment could in turn alter the composition of the extracellular matrix (ECM), now known to be affected by and influential to prostate cancer cell behavior [47].

PSA (Prostate Specific Antigen) is a protein produced by the prostate gland that has been widely used to detect prostate cancer, however, its routine and extensive use is now being questioned for several reasons. First, total PSA levels in blood can result in false negatives where men who have prostate cancer do not have elevated PSA. In addition to this, there are some non-cancerous conditions that lead to increased PSA, such as prostatitis (inflammation of the prostate) or prostatic hyperplasia (BPH), leading to false positives. Compounding this problem is the fact that the actual “normal” levels of PSA are not clearly known and, furthermore, the normal PSA range can vary with age and race. The ultimate harm in the current PSA testing, therefore, is that men will either go untreated (in the case of false negatives) or unnecessarily be treated (in the case of false positives), leading to complications with harmful side effects. Indeed only 25% of men who have a prostate biopsy due to elevated PSA actually have prostate cancer [48]. There is a need, therefore, for improved biomarkers for prostate cancer detection that could, for example, predict different stages of prostate cancer or, even to discriminate those that are predicted to progress compared to those that will remain benign. The use of WISP1 and even some of the other CCN family members offer new candidates that could be further tested for this purpose.

Prostate cancer is the 2nd leading cause of cancer-related death among men [2]. It is generally thought to occur as an androgen-dependent tumor that can progress to a highly invasive androgen-independent tumor. When the disease is advanced, the tumor continues to proliferate, spread locally, and metastasizes to lymph nodes and bone. At this point, the disease is incurable. Targeted antibody therapy has proven efficacious in clinical cancer treatment, making this a reasonable approach to consider for the development of new therapies for prostate cancer. During the course of our studies, we generated antibodies towards human WISP1 protein and found it to be highly expressed in the stromal tissue of early stages of prostate cancer in both humans and mice. Our hypothesis was that up-regulation of WISP1 in stroma creates a hospitable niche for tumor cells that supports its growth and invasion, and final establishment in bone. As we predicted, WISP1 antibody treatments reduced both PC3 xenograft growth and cancer spread to bone in mice. Taken together our new findings provide a promising foundation for future development of diagnostics and therapeutics and based on WISP1 detection and neutralization.

## Supporting Information

File S1 **Specificity of anti-WISP1/CCN4 when probed for Cyr61/CCN1, CTGF/CCN2 and Nov/CCN3.** **A.** Alignment of the human and mouse sequence of WISP1 showing the position and sequence of the peptides used to generate antibodies LF-185 (grey box) and LF-187 (black box). Amino acids that are identical between mouse and human are shown on the line between the human and mouse sequences,+indicates sequences that are similar but not identical between the two species and gaps are created for best alignment. **B.** Western blot containing 20 ng/lane of purified Cyr61/CCN1, CTGF/CCN2, Nov/CCN3 or WISP1/CCN4 probed with LF-185. **C.** Western blot containing 20 ng/lane of purified Cyr61/CCN1, CTGF/CCN2, Nov/CCN3 or WISP1/CCN4 probed with LF-187. Molecular weight markers are show to the left of the blots.
(TIF)

File S2 **Representative picture of metastasis of PC3-Luc cells 1 and 4 weeks after intracardiac injection in mice treated with either PBS, IgG or WISP1 antibodies.** A consistent site of establishment was the head/jaw/snout with other sites affected including the femur and spine.
(TIF)

File S3 **BMD ratio before and after treatments. DEXA scans of mice showing the Bone Mineral Density (BMD) in mice treated with PBS, IgG or anti-WISP1.** No significant differences were detected between the experimental groups.
(TIF)

File S4 **Tumor weight calculated from caliper measurements over time.** Measurements were taken at 4 and 6 weeks of treatment with PBS, IgG (control) or anti-WISP1 (LF-185).
(TIF)

File S5 **WISP1 expression in PC3-Luc tumors and cultured cells. A.** Diagram describing control experiments performed to examine the chemotaxis capacity of PC3-Luc cells using FBS (fetal bovine serum). **B.** Quantitation of the levels of cell migration in each of the parameters outlined in panel A. ***p<0.001.
(TIF)

File S6 **Control experiment showing specific chemotaxis of PC3 cells. A.** Immunohistochemistry of sections through xenografts of PC3-Luc sub-cutaneous tumors stained with WISP1 antibodies (left panel or IgG (right panel). **B**. RT-PCR of mRNA extracted from cultured PC3-Luc cells amplified using oligonucleotides specific for human WISP1. Left lane, marker, middle lane, 18S control, right lane WISP1.
(TIF)
