# Supplementary figures and images for: WISP1/CCN4: A Potential Target for Inhibiting Prostate Cancer Growth and Spread to Bone
Source: PLoS One. 2013 Aug 14;8(8):e71709. doi: 10.1371/journal.pone.0071709 (PMC3743748; doi:10.1371/journal.pone.0071709)

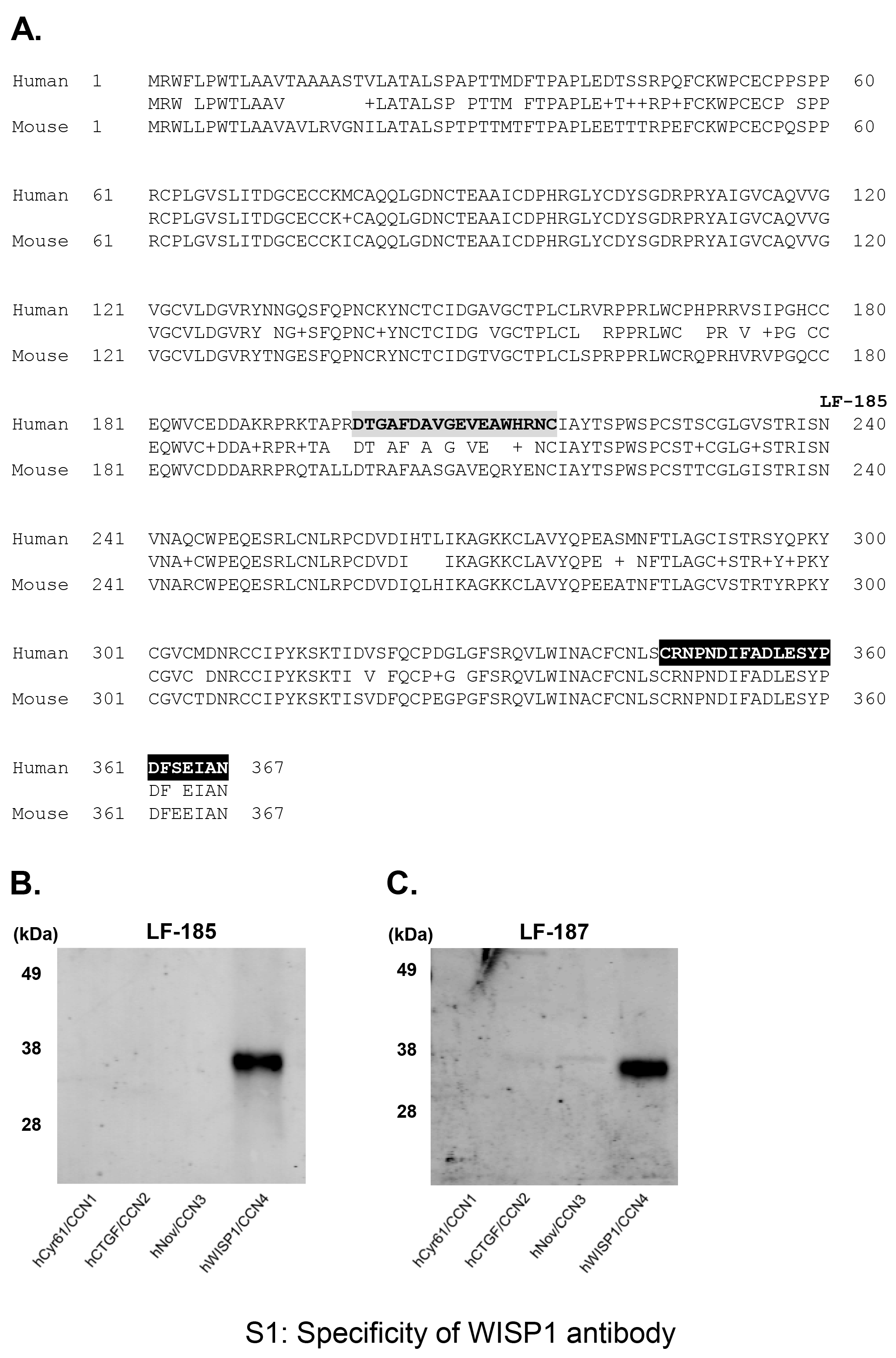

Supplement: File S1 — Specificity of anti-WISP1/CCN4 when probed for Cyr61/CCN1, CTGF/CCN2 and Nov/CCN3. A. Alignment of the human and mouse sequence of WISP1 showing the position and sequence of the peptides used to generate antibodies LF-185 (grey box) and LF-187 (black box). Amino acids that are identical between mouse and human are shown on the line between the human and mouse sequences,+indicates sequences that are similar but not identical between the two species and gaps are created for best alignment. B. Western blot containing 20 ng/lane of purified Cyr61/CCN1, CTGF/CCN2, Nov/CCN3 or WISP1/CCN4 probed with LF-185. C. Western blot containing 20 ng/lane of purified Cyr61/CCN1, CTGF/CCN2, Nov/CCN3 or WISP1/CCN4 probed with LF-187. Molecular weight markers are show to the left of the blots. (TIF) [file pone.0071709.s001.tif]

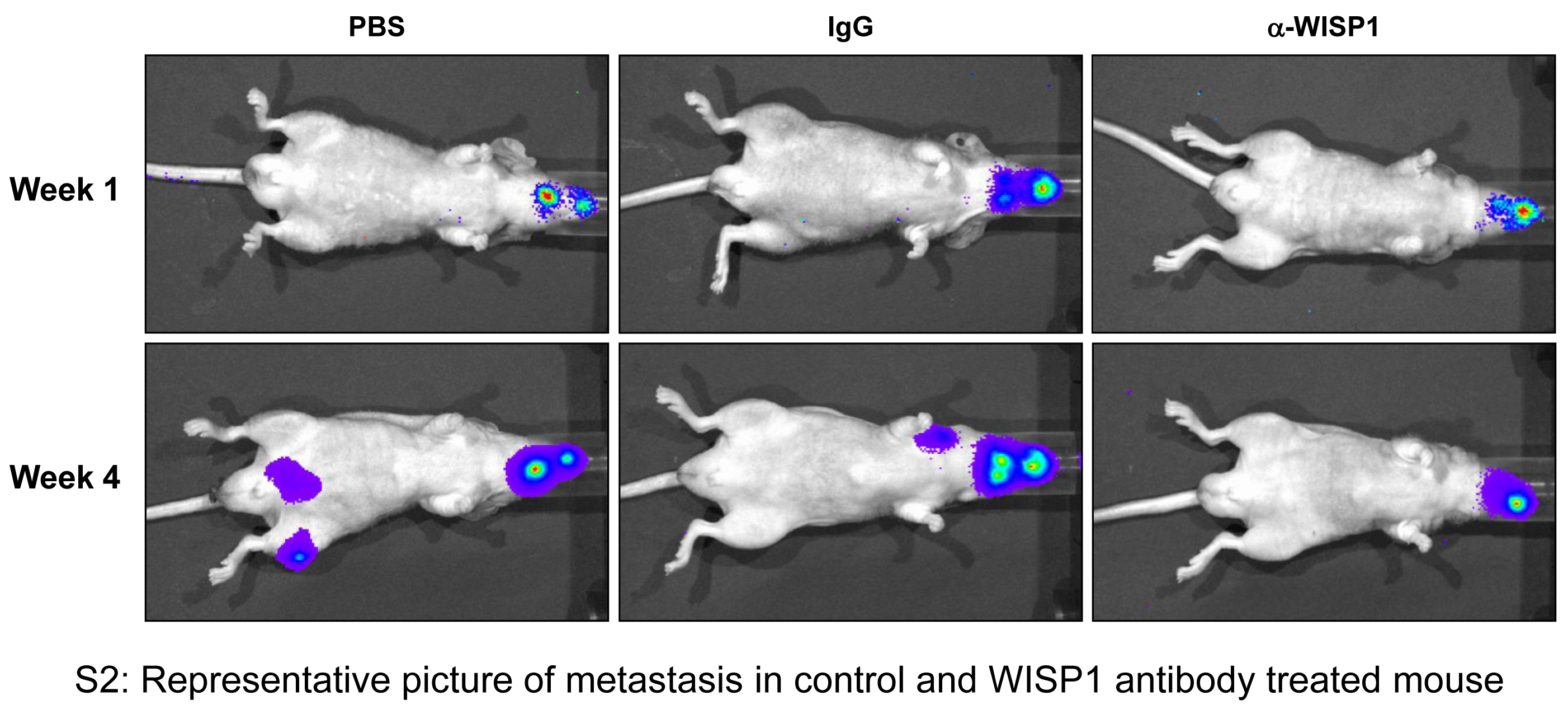

Supplement: File S2 — Representative picture of metastasis of PC3-Luc cells 1 and 4 weeks after intracardiac injection in mice treated with either PBS, IgG or WISP1 antibodies. A consistent site of establishment was the head/jaw/snout with other sites affected including the femur and spine. (TIF) [file pone.0071709.s002.tif]

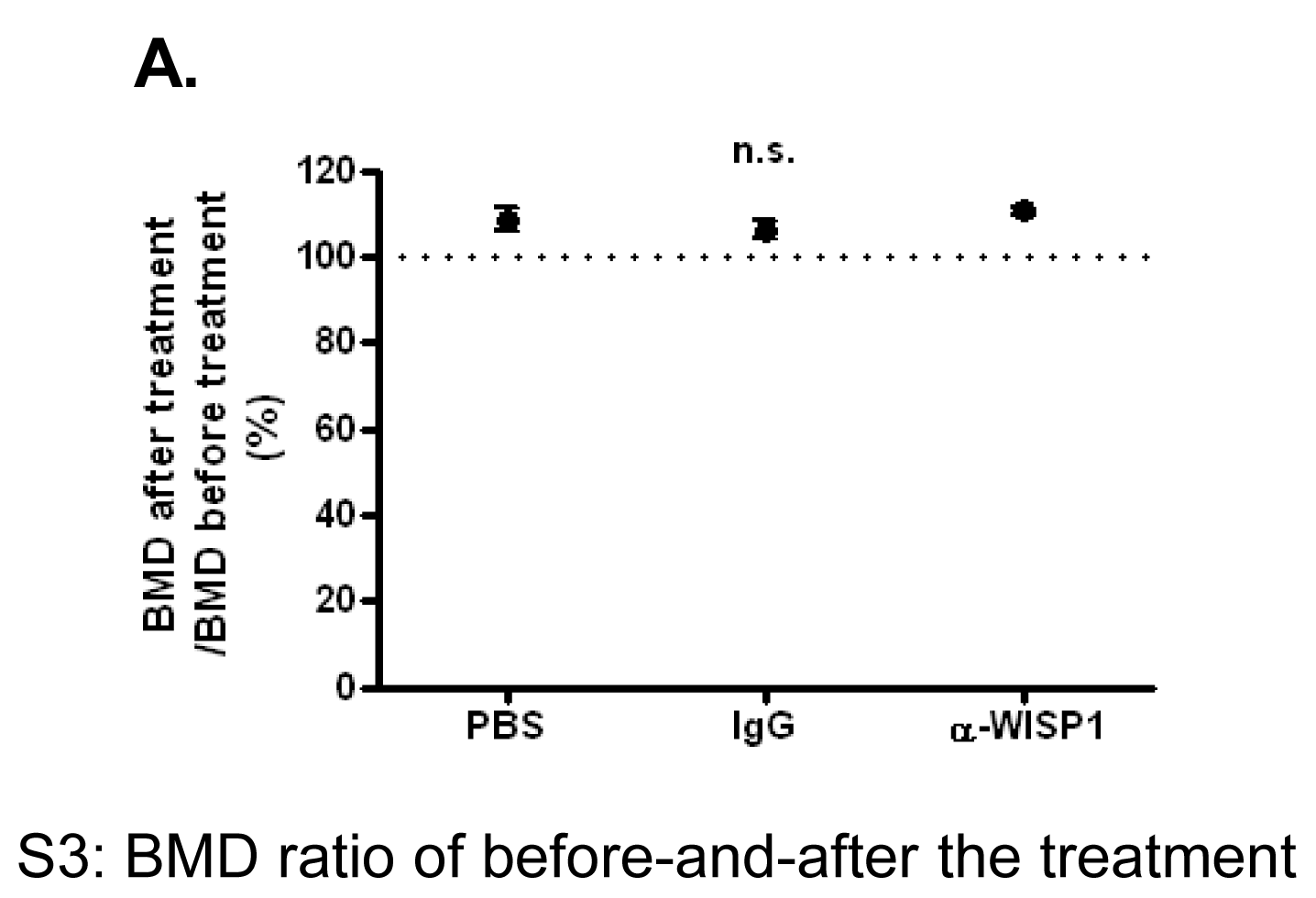

Supplement: File S3 — BMD ratio before and after treatments. DEXA scans of mice showing the Bone Mineral Density (BMD) in mice treated with PBS, IgG or anti-WISP1. No significant differences were detected between the experimental groups. (TIF) [file pone.0071709.s003.tif]

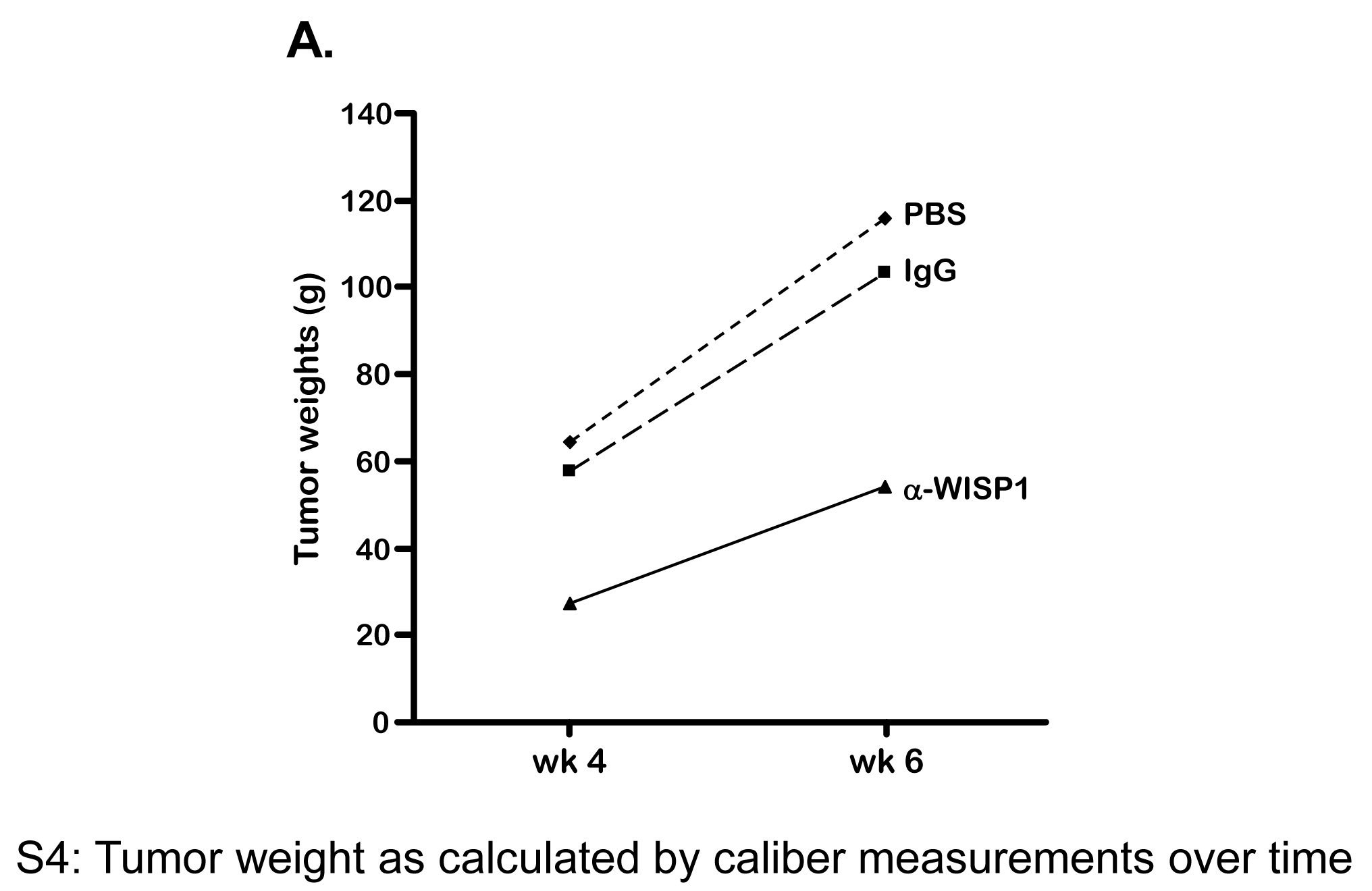

Supplement: File S4 — Tumor weight calculated from caliper measurements over time. Measurements were taken at 4 and 6 weeks of treatment with PBS, IgG (control) or anti-WISP1 (LF-185). (TIF) [file pone.0071709.s004.tif]

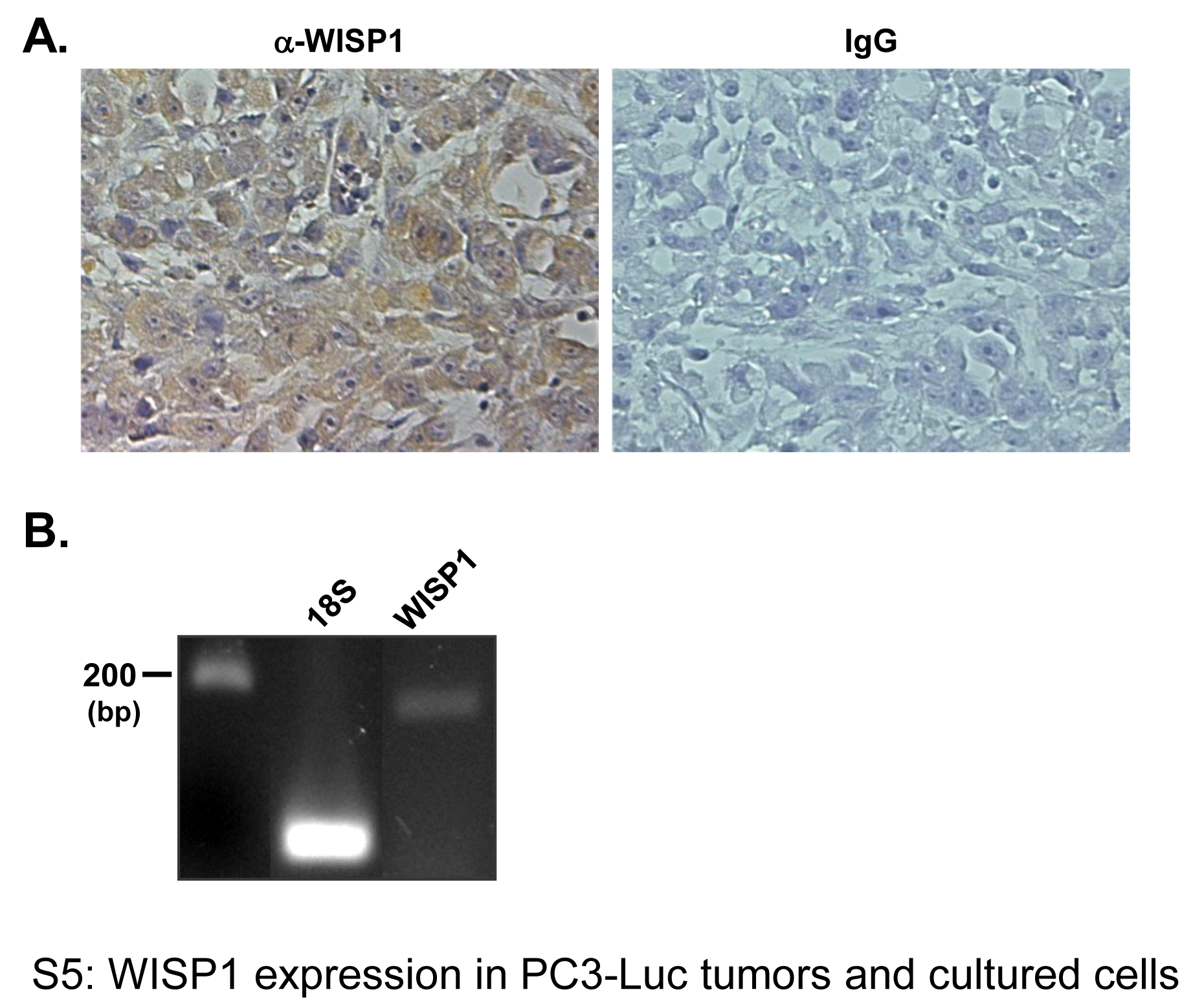

Supplement: File S5 — WISP1 expression in PC3-Luc tumors and cultured cells. A. Diagram describing control experiments performed to examine the chemotaxis capacity of PC3-Luc cells using FBS (fetal bovine serum). B. Quantitation of the levels of cell migration in each of the parameters outlined in panel A. ***p<0.001. (TIF) [file pone.0071709.s005.tif]

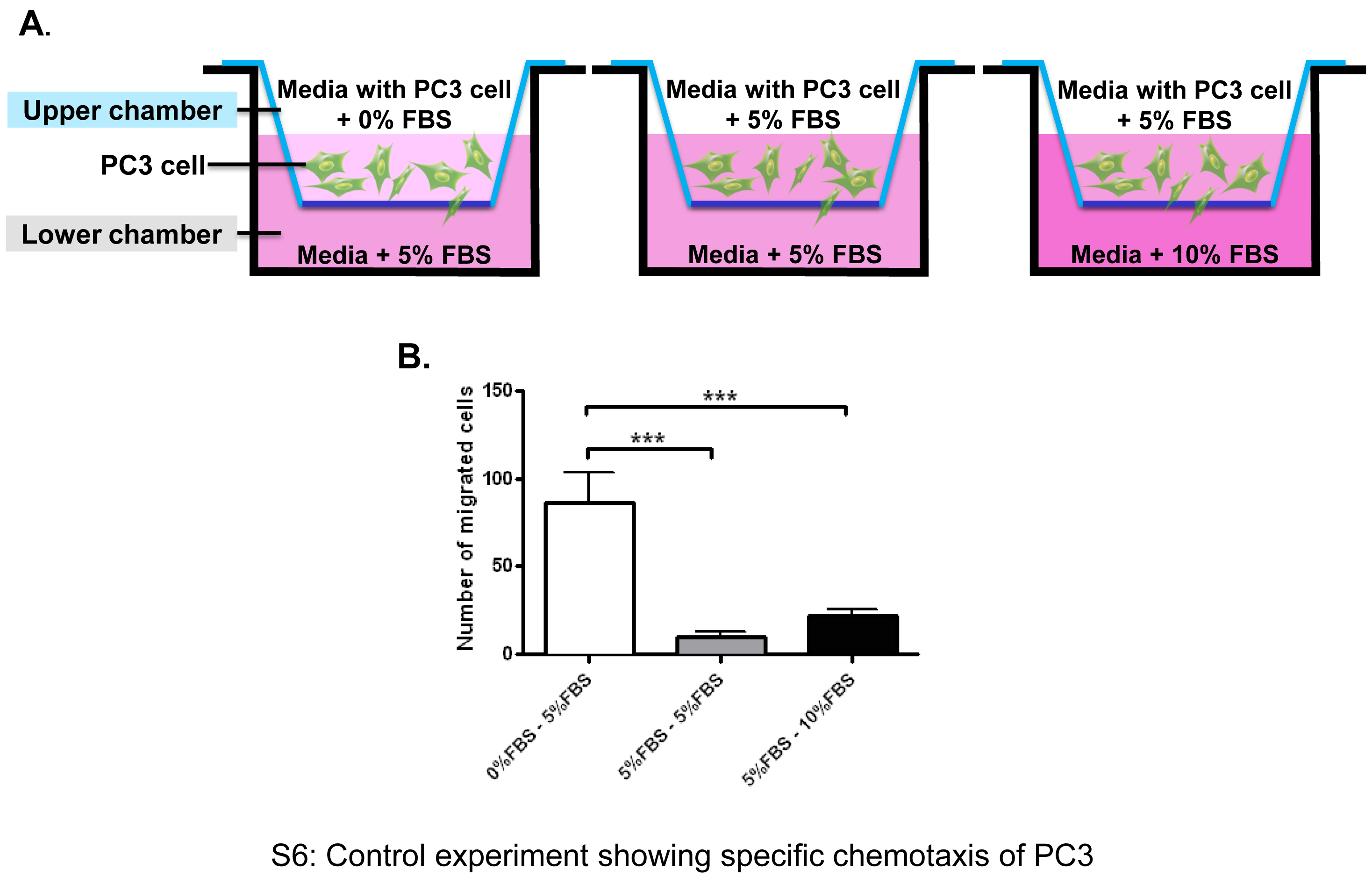

Supplement: File S6 — Control experiment showing specific chemotaxis of PC3 cells. A. Immunohistochemistry of sections through xenografts of PC3-Luc sub-cutaneous tumors stained with WISP1 antibodies (left panel or IgG (right panel). B. RT-PCR of mRNA extracted from cultured PC3-Luc cells amplified using oligonucleotides specific for human WISP1. Left lane, marker, middle lane, 18S control, right lane WISP1. (TIF) [file pone.0071709.s006.tif]
